# Resveratrol Anti-Obesity Effects: Rapid Inhibition of Adipocyte Glucose Utilization

**DOI:** 10.3390/antiox8030074

**Published:** 2019-03-26

**Authors:** Christian Carpéné, Francisco Les, Guillermo Cásedas, Cécile Peiro, Jessica Fontaine, Alice Chaplin, Josep Mercader, Víctor López

**Affiliations:** 1 Institute of Metabolic and Cardiovascular Diseases, INSERM, UMR1048, Team 1, 31342 Toulouse, France; jessica.fontaine@inserm.fr; 2 I2MC, University of Toulouse, UMR1048, Paul Sabatier University, 31432 Toulouse, CEDEX 4, France; cecile.peiro@univ-tlse3.fr; 3 Department of Pharmacy, Faculty of Health Sciences, Universidad San Jorge, 50830 Villanueva de Gállego (Zaragoza), Spain; fles@usj.es (F.L.); guille.farm11@gmail.com (G.C.); ilopez@usj.es (V.L.); 4 Instituto Agroalimentario de Aragón-IA2, CITA-Universidad de Zaragoza, 50013 Zaragoza, Spain; 5 Cardiovascular Research Institute, School of Medicine, Case Western Reserve University, Cleveland, OH 44106, USA; alice.chaplins@gmail.com; 6 Department of Fundamental Biology and Health Sciences, University of the Balearic Islands, 07122 Palma, Spain; josep.mercader@uib.es; 7 Balearic Islands Health Research Institute (IdISBa), 07122 Palma, Spain

**Keywords:** adipose cells, obesity, amine oxidases, hydrogen peroxide, polyphenols, lipogenesis

## Abstract

Studies in animal models of diabetes and obesity have shown that resveratrol mitigates complications of metabolic diseases, beyond those resulting from oxidative stress. Furthermore, results obtained with cultured preadipocytes have also revealed that prolonged resveratrol treatment impairs adipogenesis. Considering the role of adipocytes in the hypertrophy of fat stores, and keeping in mind that insulin is the main trigger of excessive energy storage during post-prandial periods, the present study aimed to investigate how short-term effects of resveratrol can limit glucose disposal in a gut-adipose tissue axis. We found that resveratrol exhibits a more potent inhibitory capacity towards α-glucosidase than pancreatic lipase activity. Resveratrol also rapidly blunts glucose transport in mature fat cells by counteracting the effect of insulin and insulin-like lipogenic agents. Within two hours, resveratrol also inhibited the incorporation of glucose into lipids of adipocytes, which was unaffected by membrane cholesterol depletion. Moreover, the comparison between adipocytes with invalidated semicarbazide-sensitive amine oxidase activity and their control, or between resveratrol and several inhibitors, did not indicate that the recently described interaction of resveratrol with amine oxidases was involved in its antilipogenic effect. Caffeine and piceatannol, previously said to interact with glucose carriers, also inhibit lipogenesis in adipocytes, whereas other antioxidant phytochemicals do not reproduce such an antilipogenic effect. This study highlights the diverse first steps by which resveratrol impairs excessive fat accumulation, indicating that this natural molecule and its derivatives deserve further studies to develop their potential anti-obesity properties.

## 1. Introduction

The potential benefits of resveratrol in treating metabolic diseases have been claimed by numerous researchers in the past decade (for reviews, see [1,2,3]). Unfortunately, the observations made on animal models of obesity have resulted in poorly effective clinical applications of resveratrol [4,5,6]. At the same time, the recently developed incretin receptor dual agonists, mimicking both glucagon-like peptide 1 and glucose-dependent insulinotropic peptide effects, are successfully decreasing both hyperglycaemia and body mass in obese and diabetic patients in current clinical trials [7]. The limited bioavailability of resveratrol (3,5,4′-trihydroxy-trans-stilbene) has been considered as a key factor responsible for such disappointing translation from basic knowledge to patient treatment [8,9,10]. Nevertheless, resveratrol is still one of the most widely studied polyphenols in the field of obesity and diabetes [11]. Although an increasing number of studies deal with the anti-obesity properties of other phytochemicals, which are expected to be more successfully extrapolated to humans, resveratrol remains a reference regarding the beneficial effects of polyphenols for treating metabolic diseases. Resveratrol effects have also been widely studied in the context of anti-inflammatory and anti-cancer prevention though its mechanism of action has not been completely deciphered [12]. Indeed, the effects of resveratrol are beyond those of other chemical antioxidants and the list of its cellular targets is far from being finalized. Thus, the present study aimed to further describe the impact of resveratrol on fuel metabolism regulation by focusing on the glucose flow from dietary carbohydrate ingestion to storage in adipose tissue. It is clear that resveratrol not only affects host metabolism by targeting adipose tissue, since this phytochemical interacts with the metabolic activities of skeletal muscle, liver [13], and intestinal microbiota [3,10]. However, it is known that the transcriptional activity of the cells present in the adipose tissue involved in energy storage management or inflammation is influenced by resveratrol [6]. Numerous studies have unanimously reported that obese mice treated with resveratrol exhibit down-regulated mRNA expression of genes related to the lipogenic pathway (including fatty acid synthase *Fas*) and decreased activity of enzymes involved in fatty acid synthesis and triacylglycerol accumulation in adipose tissue [14,15,16,17]. Similarly, in cultured preadipose cells, the transcriptional effects of resveratrol, aiming to limit adipogenesis through an increased mitochondrial function, have been evidenced by converging studies [18,19,20,21]. However, the short- and mid-term effects of resveratrol on glucose disposal during post-prandial periods remain less defined. Indeed, de novo lipogenesis as well as fatty acid re-esterification are major means for lipid deposition in fat stores exquisitely regulated by many factors (including insulin) that modulate glucose incorporation into triacylglycerols in a relatively rapid manner by modifying enzyme activities. Thus, alongside nutrigenomic regulations and hormonal control of transcription, such short-term post-translational adaptations essentially occur during post-prandial states in order to facilitate lipid storage, while they also apply for lipid mobilisation, in a mirrored manner, when adipocyte lipolysis is activated in response to stress, exercise, or fasting. In the present work, special attention was therefore paid to study the short-term influence of resveratrol on glucose handling by adipocytes.

In view of our previous observations on the capacity of resveratrol to reduce the measured levels of hydrogen peroxide in human adipose tissue [22,23] or to modulate triacylglycerol breakdown [24], our working hypothesis was that resveratrol reduces excessive fat accumulation by limiting glucose supply to adipocytes. In fact, resveratrol interacts with glucose transporters (GLUTs), including the ubiquitous human GLUT1 [25], and impairs insulin-stimulated GLUT4 translocation and expression in 3T3-L1 adipocytes [19,26]. This prompted us to determine glucose uptake and metabolism in fat cells under the short-term influence of resveratrol. Moreover, resveratrol inhibits monoamine oxidase (MAO) [23,27], a hydrogen peroxide-generating enzyme that is expressed in adipocytes alongside another enzyme involved in oxidative deamination, the semicarbazide-sensitive oxidase (SSAO). Considering we have previously reported that phenelzine, a MAO and SSAO inhibitor, inhibits lipogenesis in mouse fat cells [28] in an apparently similar manner to that of resveratrol [29], it appeared crucial to test resveratrol in fat cells with invalidated SSAO and to compare its antilipogenic effect to several related pharmacologic agents. Furthermore, the direct influence of a cholesterol depleting agent was tested on the resveratrol antilipogenic action in mouse fat cells, since resveratrol lowers cholesterol levels in blood and liver of obese rodents [15,16,17], and cholesterol depletion affects insulin sensitivity of adipocytes [30,31].

Lastly, α-glucosidase and lipase are key enzymes involved in the intestinal hydrolysis of dietary carbohydrates and fat, respectively. While α-glucosidase breaks down starch and disaccharides to glucose and monosaccharides, pancreatic lipase rapidly converts a triacylglycerol to a monoacylglycerol and two free fatty acids. The suppression of the absorption of triacylglycerols and carbohydrates by inhibitors of these enzymes is instrumental in preventing obesity. The capacities of resveratrol in inhibiting these two enzymes were therefore assessed, since they might influence the resulting hexoses available for adipocytes.

We therefore aimed to confirm whether resveratrol can modify the glucose supply along a gut-adipose tissue axis, by determining its capacity to: (1) Inhibit glucose production by intestinal α-glucosidase; (2) impair glucose uptake in mouse adipocytes; and (3) limit subsequent glucose incorporation into lipid stores.

## 2. Materials and Methods

### 2.1. Animals

Swiss mice and C57BL/6 mice (Charles River, L’Arbresle, France) were bred according to INSERM guidelines for animal care (Institut National de la Santé et de la Recherche Médicale, France; Permission Number: C31 55507). Animal procedures were approved on 20 March, 2012, with code 12-1048-03-15, by the Animal Ethics Committee of the unit US006 CREFRE (Centre Régional d’Exploration Fonctionnelle et Ressources Expérimentales, Toulouse, France).

Mice expressing a mutated SSAO, lacking oxidase activity (knock-in line called AOC3KI), were obtained after homologous recombination in embryonic stem cells with an *Aoc3* carrying Y471F point mutation, blastocyst injection, and crossing for in vivo Cre excision. Offspring were backcrossed onto a C57BL/6 genetic background by genOway (Lyon, France) and those with congenic homozygous knock-in were kindly given by Smith (BioTie Ther., Turku, Finland). No tissular SSAO activity was found in mice from this AOC3KI lineage, while present in wild type (WT) [32]. Males and females were fed a standard rodent diet and sacrificed at the age of 28 weeks for adipocyte preparation and lipogenesis assays, as described below.

In addition, a total of 20 male Swiss mice were used for adipocyte preparation to measure hexose uptake.

### 2.2. Adipocyte Preparation and Glucose Transport Assays

Adipocytes were isolated from visceral and inguinal fat pads, as recently described [33]. Briefly, fat depots were removed and digested by 15 µg/mL type TM liberase (Roche Diagnostics, Meylan, France) at 37 °C in Krebs–Ringer buffer pH 7.4, containing 15 mM bicarbonate, 10 mM HEPES, 5.5 mM glucose, and 3.5% of bovine serum albumin (KRBH buffer). Digestion was followed by filtration of the buoyant adipocytes with pieces of nylon stockings, and two washes in KRBH buffer. To investigate the effect of resveratrol on glucose transport, the radiometric method based on the uptake of the non-metabolizable [^3^H]-2-deoxyglucose during 10 min incubation, previously described for human fat cells [34], was used for Swiss mouse adipocytes.

### 2.3. Lipogenesis in Isolated Adipocytes

Lipogenic activity was determined by measuring the radioactivity incorporated from d–[3–^3^H]–glucose (Perkin Elmer, Waltham, MA, USA) into cellular lipids in adipocytes from C57BL/6 mice. Briefly, slight adaptations of the original radiometric insulin bioassay developed by Moody and co-workers [35] allowed us to determine the incorporation of 0.6 mM radioactive glucose into lipids during 120 min incubation, as previously described [33]. For each tested condition, the same vial was used for incubation at 37 °C, lipid extraction in a liquid scintillation cocktail for non-aqueous samples (InstaFluor–Plus, PerkinElmer, Waltham, MA, USA), and counting of the labelled neo-synthesized lipids.

### 2.4. Anti-Adipogenic Effect of Resveratrol on Cultured Preadipocytes

The 3T3 F442A cells were grown at 37 °C under 5% CO_2_ in DMEM, supplemented with 10% foetal calf serum and antibiotic mixture (100 U/mL penicillin + 100 µg/mL streptomycin) until confluence. Then, cells were induced into adipocyte differentiation by 50 nM insulin for 8 days without (positive control for optimal differentiation) or with 20 µM resveratrol, under already described culture conditions [36].

### 2.5. α-Glucosidase and Lipase Inhibition

The α-glucosidase activity was evaluated using a 96-microplate reader, based on a method using α-glucosidase from *Saccharomyces cerevisiae*, as already described [37]. Each well contained 100 μL α-glucosidase (1.0 U/mL) with 50 μL of resveratrol, the reference inhibitor acarbose, or ascorbic acid at the final indicated concentrations. After preincubation for 10 min, 50 μL of 4-nitrophenyl α-d-glucopyranoside (dissolved in 20 mM phosphate buffer to reach 3.0 mM, pH 6.9) was added to start the reaction for 20 min at 37 °C. Absorbance was measured at 405 nm.

Lipase inhibition was measured using a previously published protocol [38] with modifications required for the use of the 96-microplate reader. Each well contained resveratrol and 40 μL of lipase type II from porcine pancreas at 2.5 mg/mL, prepared in 100 mM Tris and 5 mM CaCl_2_, pH 7.0. After 15 min preincubation, 20 μL of 10 mM p-nitrophenyl butyrate solution was added to each well for another 15 min incubation at 37 °C. Absorbance was read at 405 nm, and orlistat was used as a reference inhibitor.

### 2.6. Chemicals

Trans-resveratrol, insulin, benzylamine, and other reagents were obtained from Sigma–Aldrich–Merck (Darmstadt, Germany), except as otherwise stated. Resveratrol was dissolved in dimethyl sulfoxide (DMSO, from Sigma–Aldrich) according to [23], briefly giving a final concentration of 89 mM (0.6% *v*/*v*) when present together with resveratrol at a dose of 100 µM. The same vehicle was used for methyl-β-cyclodextrin, kindly provided by Prof. Delmas (University of Burgundy, Dijon, France).

### 2.7. Statistical Analysis

Experimental data are given as mean ± SEM of the indicated number of independent observations and were analysed with IBM SPSS Statistics, version 25.0 (IBM Corp, Armonk, NY, USA) by one-way ANOVA or paired Student’s *t*-test when indicated. Statistical significance was assumed when *p* < 0.05.

## 3. Results

### 3.1. In Vitro Inhibition of α-Glucosidase and Lipase Activity by Resveratrol

**Figure 1:**
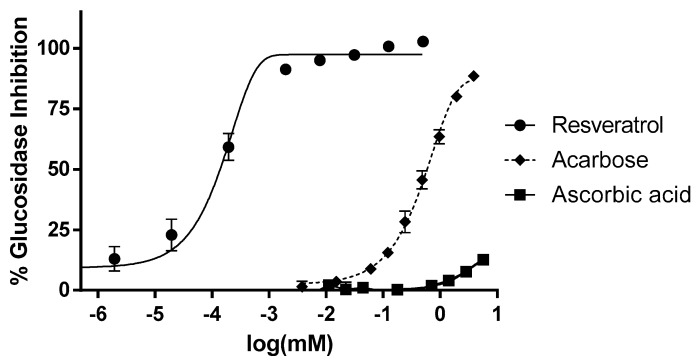
α-Glucosidase activity is dose-dependently inhibited by resveratrol. The *x* axis represents the logarithm of compound concentration in mM. Tested concentrations of resveratrol were in the range of 2 × 10^−7^–0.5 mM. IC_50_ were calculated by non-linear regression, using GraphPad Prism 6. Acarbose was tested as a positive control drug in the range of 0.004–3.9 mM. Ascorbic acid (vitamin C) was tested as an antioxidant reference in the range of 0.005–5.7 mM. All concentrations were tested at least in triplicate, each point representing mean ± SEM.

In order to test the effect of resveratrol on α-glucosidase activity, different concentrations (from 2 nM to 0.5 mM) of trans-resveratrol were added to a solution containing α-glucosidase (1.0 U/mL) for 20 min. As shown in Figure 1, resveratrol dose-dependently inhibited α-glucosidase catalytic activity at much lower doses than the reference inhibitor acarbose. The two sigmoid dose-dependent curves were separated by more than three orders of magnitude. The IC_50_ values (concentration of inhibitor decreased by half the response measured) were 0.1 µM for resveratrol and 590 µM for acarbose, highlighting the dramatic difference between both compounds. In the same conditions, ascorbic acid was devoid of any notable inhibitory effect.

**Figure 2:**
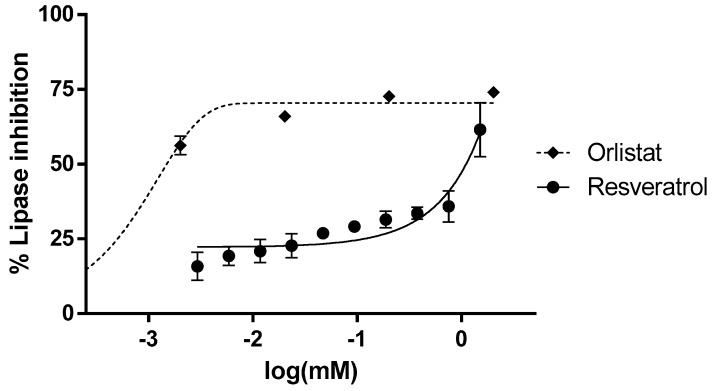
Lipase activity is dose-dependently inhibited by resveratrol. The *x* axis represents the logarithm of the concentration in mM. Tested concentrations of resveratrol were in the range of 0.003–1.5 mM. Orlistat was tested as a positive control drug in the range of 0.002–2 mM. IC_50_ were calculated by non-lineal regression, using GraphPad Prism 6. All concentrations were tested at least in triplicate, each point representing mean ± SEM.

Furthermore, in order to look into the effects of resveratrol on lipase activity, ten doses of resveratrol were added to a solution containing 2.5 mg/mL pancreatic lipase (Figure 2). Lipase was inhibited by resveratrol, especially at the highest dose (1.5 mM). However, in contrast with α-glucosidase assays, resveratrol was much less efficient than the reference inhibitor orlistat, which exhibited an IC_50_ value of 1.5 µM. Thus, it seems that maximal inhibition by resveratrol of lipase activity occurred only at millimolar doses of the polyphenol, which could be considered as supranutritional levels, while the α-glucosidase inhibition was more likely to be nutritionally relevant.

### 3.2. Resveratrol Inhibits Insulin-Stimulated Adipogenesis in Preadipocytes

To validate our resveratrol preparation on cellular models, we first verified whether the well-recognized anti-adipogenic effect of resveratrol [19,20,39] was confirmed in a cultured murine preadipocyte lineage. After 8 days of differentiation with 50 nM insulin, the 3T3 F442A preadipocytes accumulated 65 times more triacylglycerol than those at day 0. The daily addition of resveratrol at a final concentration of 20 µM reduced the lipid accumulation occurring during this long differentiation process by 35%: Insulin-treated wells contained 0.25 ± 0.02 g/L of triacylglycerol, while wells treated with insulin plus resveratrol contained only 0.17 ± 0.03 g/L (*n* = 6, *p* < 0.03). Such validation allowed us to continue our research on resveratrol’s “delipidating” effect, during shorter exposure on mature fat cells, by measuring a very rapid response that essentially depends on the recruitment and the intrinsic activity of glucose transporters: The uptake of non-metabolizable hexose.

### 3.3. Resveratrol Rapidly Inhibits Insulin and Benzylamine-Induced Glucose Uptake in Adipocytes

**Figure 3:**
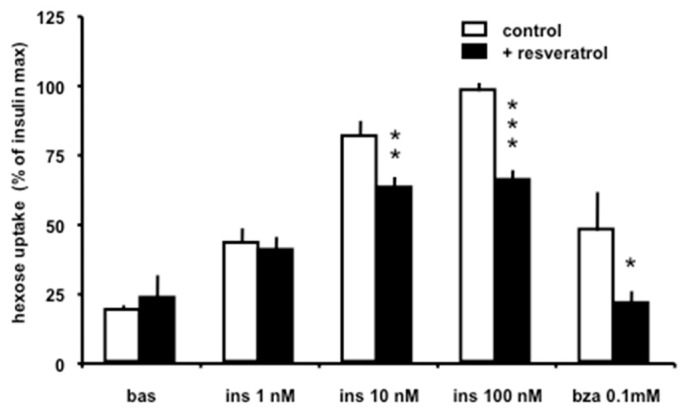
Inhibition of the insulin- or benzylamine-induced activation of glucose uptake in Swiss mouse adipocytes by resveratrol. Fat cells were incubated for 45 min without (basal, bas) or with increasing concentrations of insulin (ins), or with 100 µM benzylamine (bza), in the absence (control) or in the presence of 100 µM trans-resveratrol (plus resveratrol, black columns). Data are expressed as the percentage of the maximal stimulation of glucose uptake by 100 nM insulin alone, which was not altered by the DMSO vehicle present in control conditions (open columns). Each column is the mean + SEM of 8–16 observations. Significantly different from corresponding control at: * *p* < 0.05; ** *p* < 0.01; *** *p* < 0.001, by paired *t* test.

Since glucose transport is the first step of glucose utilization by fat cells and is regulated by insulin within minutes, it was verified whether resveratrol influences the insulin stimulation of 2-deoxyglucose uptake in mouse adipocytes. As shown in Figure 3, incubation of Swiss mouse adipocytes with resveratrol significantly limited the insulin-derived maximal activation of hexose uptake, assayed in 10 min. The vehicle (DMSO 0.6% *v*/*v* final) used to dissolve resveratrol at a final concentration of 100 µM did not influence basal or insulin-stimulated hexose uptake. It was then tested whether the stilbenoid was impairing the insulin-like action of benzylamine. Indeed, the stimulation with 100 µM of this SSAO substrate, reaching almost half-maximal activation of hexose uptake, was impaired by 100 µM resveratrol. It has been previously demonstrated that such insulin-like action of benzylamine is dependent on its oxidation by amine oxidases expressed in adipocytes and the subsequent generation of hydrogen peroxide [40].

### 3.4. Inhibition of Lipogenesis by Resveratrol is Independent of SSAO Activity

**Figure 4:**
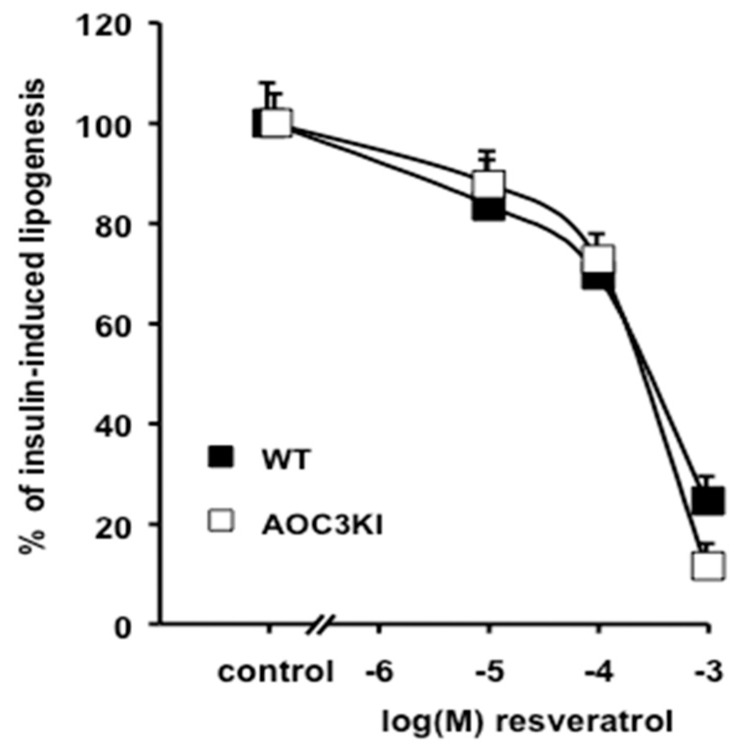
Dose-dependent inhibition of glucose incorporation into lipids of mouse adipocytes. Lipogenic activity was measured during a 120 min period, via the incorporation of ^3^H-glucose into lipids of native adipocytes from wild-type (WT, black squares) or SSAO-invalidated mice (AOC3KI, open squares). The *x* axis represents the logarithm of the molar concentration (M). Data as a percentage of 100 nM insulin activation of glucose incorporation into adipocyte lipids (control). Each point is the mean ± SEM of 5–10 preparations. No significant gene effect was detected.

One of the fates of glucose as it enters the cytoplasm of adipocytes is its conversion to fatty acids via the hexokinase phosphorylation pathway, acetyl-CoA generation, then *Fas* activation (de novo lipogenesis). Thus, it was of interest to determine the capacity of resveratrol to inhibit this process in mouse adipocytes. However, it has been shown that glucose transport activation and lipogenic stimulation by benzylamine (alone or with vanadium) are abolished in adipocytes from mice lacking the *Aoc3* gene [41]. Therefore, we compared the influence of resveratrol on lipogenesis in adipocytes from WT mice and from mice with a genetically modified *Aoc3* gene encoding for a catalytically inactive SSAO [32]. We took advantage of such *Aoc3* point-mutation knock-in mice (AOC3KI mice), devoid of catalytically active SSAO [32] to perform lipogenic tests in SSAO-deficient adipocytes. As previously reported, insulin, but not benzylamine, is active in stimulating glucose utilization in these adipocytes [41,42]. We did not find any detectable alteration of the resveratrol inhibitory action on adipocyte lipogenesis in AOC3KI mice (Figure 4). Since resveratrol triggered a dose-dependent antilipogenic response, even in the absence of a functional SSAO, it can be deduced that such amine oxidase activity was not involved in the observed effects of resveratrol.

Although the SSAO, highly expressed at the cell surface of fat cells, was not required for the antilipogenic effect of resveratrol, we verified whether other membrane events were involved in the non-transcriptional effects of the stilbenoid. We hypothesized that depleting cholesterol and disrupting lipid rafts with an agent such as methyl-β-cyclodextrin, already used to study insulin sensitivity of adipocytes [30], could affect the resveratrol inhibition of glucose incorporation into lipids.

**Figure 5:**
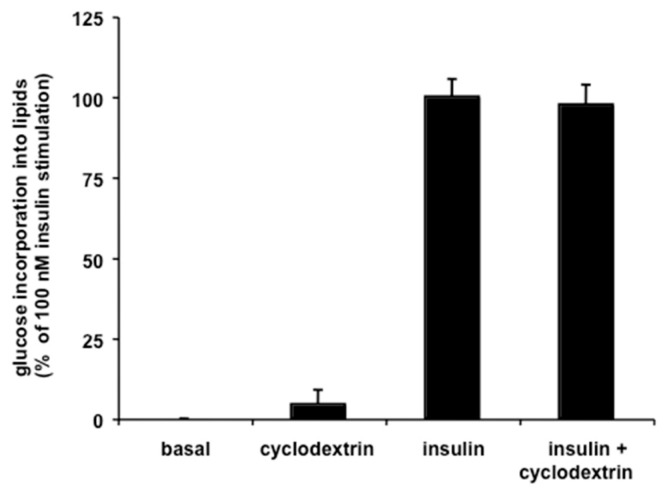
Lipogenic activity was measured during 120 min with adipocytes freshly isolated from mouse intra-abdominal fat depots. Final concentration of insulin was 100 nM, while methyl-β-cyclodextrin was tested at 1 mM. In C57Bl6 mouse adipocytes, the insulin effect was equivalent to a 2.7 ± 0.2-fold increase over basal lipogenesis. Lipogenesis was expressed as a percentage of maximal insulin-induced activation, with basal values set at 0%. Mean ± SEM of 13–20 preparations.

Preliminary experiments indicated that methyl-β-cyclodextrin treatment did not produce significant effect on basal- or insulin-stimulated lipogenesis (Figure 5). It was then tested whether the presence of this cholesterol-depleting agent [30,31] was sufficient to alter the resveratrol-induced inhibition of glucose incorporation into lipids.

**Figure 6:**
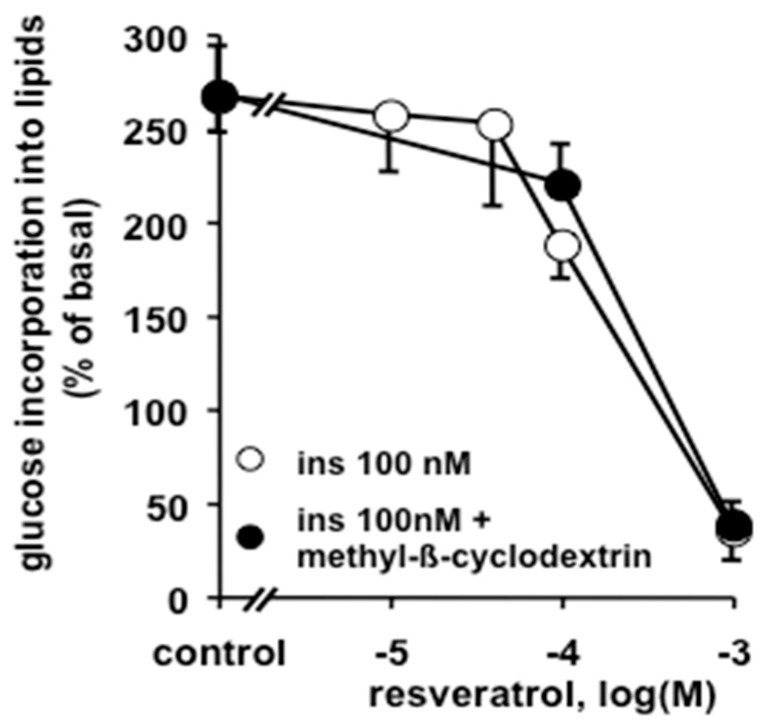
Inhibition of mouse adipocyte lipogenesis by resveratrol is not influenced by cholesterol depletion. Incorporation of ^3^H-glucose into adipocyte lipids was measured after 120 min with 100 nM insulin, 1 mM methyl-β-cyclodextrin, and the indicated increasing doses of resveratrol. Data were expressed as percentage of basal lipogenesis, set at 100%. The *x* axis represents the logarithm of resveratrol concentration in M. Each point is mean ± SEM of at least 10 preparations. No significant effect of methyl-β-cyclodextrin (black circles) was found on resveratrol inhibition of insulin-stimulated lipogenesis (open circles).

At 100 µM, resveratrol impaired approximately one third of insulin stimulation, while at 1 mM, it abolished almost all lipogenic activity, limiting glucose utilisation largely below basal levels, irrespective of the presence or absence of methyl-β-cyclodextrin (Figure 6).

### 3.5. Comparison of the Antilipogenic Effects of Resveratrol and Other Agents

We further compared the antilipogenic activity of resveratrol to other antioxidant agents or molecules supposed to interact with glucose uptake, glycerophosphate synthesis, or triacylglycerol accumulation. Table 1 clearly indicates that antioxidants such as ascorbic acid or *N*-acetylcysteine were not antilipogenic, even at 1 mM. This finding does not support that the antioxidant properties of resveratrol were sufficient to impair fat accumulation. The sigma receptor agonist opipramol, which shares neuroprotective properties with resveratrol [43], was inefficient in acutely limiting insulin-stimulated glucose incorporation into lipids, as was the case for the NADPH-oxidase inhibitor apocynin, known to prevent oxidative stress in obesity models [44] (Table 1). The phosphatase inhibitor, sodium orthovanadate, did not alter insulin maximal action (Table 1), while when used alone, it mimicked half of the insulin lipogenic effect (not shown). Phenelzine, which inhibits both MAO and SSAO, was antilipogenic as previously described [28]. Among the tested compounds, caffeine, known for its interaction with phosphodiesterases, adenosine A2 receptors, and glucose carriers, was the sole natural non-phenolic compound capable of reproducing the resveratrol inhibitory effect on glucose incorporation into fat cells. Since caffeine inhibits human MAO activity [45], it appeared logical to test whether pargyline, a reference MAO inhibitor, was antilipogenic. However, the weak tendency of pargyline in counteracting insulin stimulation did not prompt us to further test whether MAO inhibition, one of the multiple properties recently described for resveratrol [22,23,27], was primarily involved in its short-term antilipogenic effect on adipocytes. Lastly, piceatannol, which is a hydroxylated resveratrol known to exert anti-obesity effects [46,47], exhibit antioxidant properties [48], inhibit MAO [23,27], and to interact with GLUT translocation [49], was also clearly as antilipogenic as resveratrol.

## 4. Discussion

Our approach did not allow for deciphering the exact mechanisms by which resveratrol prevents fat accumulation in treated obese rodents. However, our observations support that various steps involved in the handling of ingested carbohydrates are rapidly inhibited by resveratrol in an integrated manner along the intestine–adipose tissue axis, leading to limited fat deposition. The direct inhibition of α-glucosidase-dependent production of glucose before its passage across the intestinal barrier, the inhibition of insulin-stimulated glucose entry into fat cells, and the inhibition of glucose incorporation into neo-synthesized lipids are therefore mechanisms likely contributing to the resveratrol anti-obesity benefits, alongside many other actions [5]. All these inhibitions of sugar handling converge to a limitation of fat cell hypertrophy already seen in animal models after prolonged supplementation with resveratrol [14,15,16,17], or reproduced in preadipocyte cultures [19,20,39]. It can be reasonably extrapolated that all these relatively rapid events also occur upon resveratrol consumption or supplementation in humans. Indeed, reduction of adipocyte size has already been reported in a resveratrol-based clinical trial [50].

In the present study, the dose of resveratrol necessary to inhibit half of the α-glucosidase activity was much lower than that required for acarbose towards yeast α-glucosidase. When studying the inhibition of mammalian α-glucosidase, Zhang et al. [51] did not find such an impressive difference between the affinity of resveratrol and that of the reference inhibitor, but concluded that the inhibition of intestinal α-glucosidase by resveratrol was likely involved in its antidiabetic effects. Moreover, these authors found that a related stilbenoid, piceatannol, similarly impaired glucose intestinal absorption by strongly inhibiting α-glucosidase activity [51]. Interestingly, we report here that piceatannol also shares with resveratrol a direct antilipogenic effect in adipocytes. Intriguingly, all these inhibitory properties appeared to occur independently of antioxidant activity, since they were not reproduced by ascorbic acid. Other polyphenols have demonstrated in vitro inhibitory properties of α-glucosidase and lipase, such as urolithin A, ellagic acid, and punicalagins from pomegranate [52] or cyanidin-3-O-glucoside from berries [53]. Metabolites from pomegranate extracts, known as urolithins, seemed to exert better inhibition profiles than acarbose, while cyanidin-3-O-glucoside showed similar activity to the antidiabetic drug. Considering all these results, we can conclude that resveratrol (IC_50_ = 0.1 µM) is more potent than urolithin A (IC_50_ = 65.7 µM) or cyanidin-3-O-glucoside (IC_50_ = 479.8 µM) in the α-glucosidase assay, revealing a better potential for biomedical applications. It can be noted that urolithin, produced during the metabolism of ellagitannins by intestinal microbiota, is not totally devoid of α-glucosidase inhibitory activity, leaving one to suppose that resveratrol metabolites also share such property. Since resveratrol has been evidenced to be largely excreted in the urine within four hours of ingestion in humans [5], its rapid actions reported here totally agree with the development of resveratrol-based pharmaceutical products or food supplements aiming to limit post-prandial hyperglycaemic excursions. During the completion of this work, a mono-glycosylated form of resveratrol, named resveratroloside, has been demonstrated to be an even stronger inhibitor of α-glucosidase activity and to limit increases in post-prandial blood glucose [54].

Resveratrol also inhibited pancreatic lipase, but with a lower potency. Such modest resveratrol action might contribute to its hypolipidemic influence [55], or to its limitation of lipid deposition in fat stores. Indeed, the latter limitation of fatty acid storage under the form of triacylglycerol has already been proposed to result mainly from an elevation of circulating levels of a fasting-induced adipocyte factor (Fiaf), a lipoprotein lipase inhibitor, seen in animals treated with resveratrol [15]. However, our results indicate that submillimolar doses of resveratrol blocked the de novo lipogenic activity in adipocytes more readily than the release of fatty acids during lipid digestion in the gut, and therefore, their uptake and re-esterification in lipid stores.

Adipocytes, together with the endothelial, immune, and stem cells found in adipose tissues, have many aspects of their metabolism regulated or even dysregulated by reactive oxygen species (ROS). The excess of nutrients may generate toxic messengers by enhancing oxidative stress, and the hypertrophied fat depots are often accompanied by chronic insulin resistance, low-grade inflammation, and a pro-inflammatory state characterizing the metabolic syndrome. Various polyphenols, such as those present in coffee, have been recognized by epidemiological approaches to mitigate such complications, by decreasing inflammation [56] or by increasing adiponectin [57], as is also the case for the flavonoids abundant in the Mediterranean diet [58,59]. Similarly, many resveratrol health benefits depend on its ability to limit ROS-induced damages, via signalling pathways involving sirtuins and forkhead box O, and by increasing the expression of diverse antioxidant enzymes in various cell types [60]. However, regarding fat cells, a member of the ROS family, namely hydrogen peroxide, has been known for a long time to mimic the insulin stimulation of glucose uptake [61]. Accordingly, benzylamine, which generates hydrogen peroxide when oxidized by SSAO in fat cells [40], facilitates glucose entry in a manner that was impaired by resveratrol. Considering the antioxidant properties of the polyphenol, it is therefore not so unexpected to observe that resveratrol inhibits in fat cells a transport activation somewhat dependent on reactive oxygen species (ROS). Nevertheless, it remains to be elucidated whether resveratrol inhibition of benzylamine action involves the direct blockade of amine oxidase activity or impairs downstream events triggered by the hydrogen peroxide produced during amine oxidation. We hypothesized that the latter inhibition was related to the antioxidant properties of resveratrol. However, various rapid inhibitory effects of resveratrol were not clearly reproduced by antioxidants: The α-glucosidase inhibition and the inhibition of insulin-stimulated glucose incorporation into lipids. These observations prompted us to test the role played by amine oxidase inhibition.

Both MAO and SSAO converge to produce hydrogen peroxide when oxidizing their substrates and mimic several of the insulin actions in adipocytes [62]. It was therefore supposed that the resveratrol antilipogenic action was mediated by their inhibition. However, the adipocytes from mice, invalidated for the *Aoc3* gene encoding SSAO, still responded to resveratrol by a notable inhibition of insulin lipogenic stimulation. This led us to consider that SSAO inhibition was not a required mechanism, necessary and sufficient for allowing the polyphenols to inhibit lipogenesis. Similarly, the MAO inhibitor pargyline did not alter the insulin-induced lipogenesis as much as resveratrol. Apparently, the recently described MAO inhibitory property of resveratrol [22,23,27] was therefore not involved in the in vitro antilipogenic effect of the stilbenoid. In fact, resveratrol has been reported as a MAO inhibitor [63], but not as a SSAO inhibitor [22], as is also the case for piceatannol [23]. On the contrary, phenelzine is a well-known MAO inhibitor that inhibits SSAO as well [64], and which shares with resveratrol and piceatannol the capacity to exert antilipogenic properties in mouse adipocytes (Carpéné et al., 2014). A further comparison of the three molecules could indicate whether MAO inhibition, SSAO inhibition, or both are required for their antilipogenic effects, or whether another common pharmacological property is responsible for this somewhat "anti-insulin" action in fat cells. Regardless, the direct limitation by resveratrol of glucose incorporation into adipocyte lipid stores, once applied in a prolonged manner, is likely contributing to the limitation of fattening, already observed in resveratrol-treated obese models [2]. Of note, there are less immune cells in white adipose tissue of SSAO-invalidated mice than in WT [65]. Thus, the similar antilipogenic effect of resveratrol in adipocytes from both genotypes suggests that the indirect anti-inflammatory effect of resveratrol is not required for a rapid limitation of glucose incorporation into fat cell lipid stores.

Among the targets of resveratrol involved in its rapid limitation of glucose utilization in fat depots, there are glucose transporters (GLUTs). Resveratrol interacts directly with such GLUTs by binding to an endofacial site and this interaction inhibits the transport of hexoses across the plasma membrane [25]. This inhibition (rapidly blunting the uptake of non-metabolizable glucose analogue) is distinct from the resveratrol effect on the intracellular phosphorylation/accumulation of glucose, also involved in the inhibition of basal and insulin-stimulated lipogenesis. In addition, since resveratrol also impairs the intestinal absorption of glucose (by inhibiting α-glucosidase), and favours glucose utilization in skeletal muscles [49,66,67], its prolonged administration results in limiting hyperglycaemia and facilitating glucose homeostasis regulation by insulin. It is therefore a "fuel repartitioning effect", leading to a redistribution of metabolic fluxes, that can explain why resveratrol is useful for anti-obesity and anti-diabetic approaches in rodents [14], rather than being a simple and deleterious anti-insulin agent. Thus, even when limited or delayed after carbohydrate ingestion, the glucose load reaching the blood flow in post-prandial periods—even if misused in part by adipocytes to synthesize fat—has a greater possibility to be used by other cell types in the body. It can be noted at this stage that the complete opposite is observed for SSAO substrates (including benzylamine), which increase the entry of glucose in adipocytes without facilitating its use in muscles (devoid of SSAO activity): They are anti-hyperglycaemic in mice, except in the SSAO-deficient ones [42], but do not reduce fat accumulation.

To further explore the specific interaction between resveratrol and adipocytes, it was tested whether its antilipogenic action was altered by the lipid raft disrupter, methyl-β-cyclodextrin. Since our attempt to produce cholesterol depletion did not notably modify resveratrol antilipogenesis, one can suggest that, apart from an impairment of hexose carrier function [68], the polyphenol also limits glucose metabolism towards lipid synthesis by inhibiting the intrinsic activity of intracellular lipogenic enzymes. In this regard, it has been reported that after chronic treatment with resveratrol, many enzymes of the lipogenic pathway are down-regulated. This is the case for the fatty acid synthase, a product of the *Fas* gene [18,69], as well as for the mRNA expression of other lipogenic enzymes: Acetyl-CoA carboxylase (*Acaca*) [15,17], phosphatidate phosphohydrolase (*Pap*) [14], and their orchestrating transcription factors (*Pparγ, Cebpα*) [13]. In this view, the role of epigenetic mechanisms (changes in microRNA and in methylation profiles) are undoubtedly involved in a sustained inhibition of lipogenesis by resveratrol action [69,70]. Thus, there is likely a co-existence of short-term and long-acting mechanisms of resveratrol to limit the excess of energy to be stored as fat, in adipose tissue, and in the liver [71], or the intestine as well [72]. Aside from such integrated limitation of lipogenesis, an activation of lipolysis cannot be excluded, but resveratrol is not a strong lipolytic agent on its own [24]; it just improves the adipocyte responses to other lipid-mobilizing factors [11].

## 5. Conclusions

Considering fat cells are apparently the most impacted cells in obesity, our investigation tested whether one of their metabolic functions, namely the synthesis of lipids, was altered by resveratrol, a promising anti-obesity natural product. Our approach confirmed that all of the antilipogenic effects of resveratrol-prolonged treatments reported in obese rodents could be summarized in short-term in vitro experiments, performed with mature adipocytes. Thus, apart from the transcriptional effects of resveratrol found in cultured preadipocytes or in the fat depots of chronically treated mice, which converge in a limitation of triacylglycerol accumulation, the polyphenol is also able to hamper, in a short-term manner, the tone of the lipogenic pathway and to limit the adipocyte responses to the insulin activation of triacylglycerol assembly. However, resveratrol, which at submillimolar doses limits insulin-stimulated glucose metabolism in adipose tissue, does not mandatorily promote hyperglycaemic reactions, since it (1) limits glucose absorption by inhibiting intestinal α-glucosidase activity at very low doses, (2) favours glucose entry in other insulin-sensitive tissues, such as skeletal muscles, and (3) is rapidly metabolized by the consumer or its gut microbiota once ingested [10].

Resveratrol can therefore be considered an agent with acute antilipogenic potential in adipocytes. Whether all the in vitro rapid actions depicted here for sub-millimolar resveratrol doses are relevant for human nutrition and adipose tissue physiology or need to be improved (e.g., by synergism with another phytochemical acting on energy dissipation or by screening more potent polyphenol metabolites/derivatives) to reach long-lasting anti-obesity effects remains to be established. Regardless, the various resveratrol actions that result in a coordinated limitation of the use of post-prandial circulating glucose by adipocytes warrant further translational research on stilbenoids, at least in the domain of the prevention of obesity- and diabetes-related complications.

## Figures and Tables

**Table 1:** In vitro effects of antioxidants, receptor agonists, or enzyme inhibitors on insulin stimulation of glucose incorporation into neosynthesized lipids of mouse adipocytes.

Agent	(*n*)	% of Insulin Lipogenic Action
Insulin 100 nM alone	(20)	100.0 ± 6.3
Ins + DMSO vehicle	(10)	97.4 ± 6.9
Ins + N-acetyl cysteine 1 mM	(9)	100.8 ± 8.4
Ins + Ascorbic acid 1 mM	(9)	89.8 ± 5.1
Ins + Opipramol 10 µM	(4)	90.9 ± 6.8
Ins + Apocynin 10 µM	(4)	99.3 ± 11.6
Ins + Pargyline 0.1 mM	(8)	91.1 ± 5.2
Ins + Phenelzine 0.1 mM	(17)	73.0 ± 4.7 *
Ins + Caffeine 1 mM	(20)	45.6 ± 2.8 ***
Ins + Piceatannol 0.1 mM	(15)	22.9 ± 2.4 ***
Ins + Resveratrol 1 mM	(8)	18.0 ± 3.4 ***

Data are given as percentages of insulin-stimulated glucose incorporation into lipids, with 100 nM insulin activating approximately four times the basal lipogenic activity after a 2 h incubation at 37 °C. Mean ± SEM of (*n*) number of observations. Ins: insulin 100 nM. Significantly different to control at: * *p* < 0.05; *** *p* < 0.001.
